# Structural abnormalities of choroid vasculature in first-episode psychosis

**DOI:** 10.1192/j.eurpsy.2023.585

**Published:** 2023-07-19

**Authors:** C. Demirlek, F. Atas, B. Yalincetin, H. B. Baykara, B. B. Akdede, M. Kaya, E. Bora

**Affiliations:** ^1^Department of Neuroscience, Dokuz Eylul University, Izmir; ^2^Department of Ophthalmology, Cerkezkoy State Hospital, Tekirdag; ^3^Department of Child and Adolescent Psychiatry; ^4^Department of Psychiatry, Dokuz Eylul University; ^5^Private Practice, Izmir, Türkiye

## Abstract

**Introduction:**

The eye is considered as a part of the central nervous system and a window of the brain. The eye and the brain, also their microvascular system, share many common features in terms of anatomy and pathophysiology. Choroid and retina related abnormalities have been reported in psychotic disorders, especially in schizophrenia. Choroidal vascularity index (CVI) is a useful tool to assess choroidal structural alterations and CVI is defined as the proportion of luminal area (LA) to the total choroidal area (TCA).

**Objectives:**

This is the first study to investigate choroidal vascularity index in first-episode psychosis (FEP) using optical coherence tomography (OCT).

**Methods:**

31 patients with FEP and 30 age and gender-matched healthy controls (HC) included in this study. All participants underwent psychiatric and ophthalmological clinical assessments. Imaging of the choroid was performed using enhanced deep imaging (EDI) protocol - spectral domain OCT. Central choroidal thickness (CCT) with EDI-OCT was measured manually from the inner border of the sclera to the outer border of the RPE vertically using the calipers of the Heidelberg reader subfoveally. The choroidal vascularity index was assessed using the EDI-OCT images.

**Results:**

There were no statistically significant differences between FEP and HC in terms of CCT (p=0.33), TCA (p=0.809) and LCA (p=0.710). The CVIs were 66.66±2.98% and 62.32±3.32% in FEP and HC, respectively and it was significantly different between groups (p<0.001, effect size d=1.38).

**Conclusions:**

These results suggest that first episode psychosis is characterized by choroidal vascular abnormalities. in vivo ophthalmological imaging with OCT, a non-invasive, fast, and safe technological tool, can be a promising biomarker to quantitatively evaluate microvasculature abnormalities in patients with psychosis.

**Disclosure of Interest:**

None Declared

